# Treatment of a Pediatric Arterial Ischemic Stroke With Tenecteplase

**DOI:** 10.7759/cureus.92560

**Published:** 2025-09-17

**Authors:** Mostafa Alsabbagh, Maham Alvi, Aryaan Khan, Alexander Venizelos

**Affiliations:** 1 Internal Medicine, Summa Health, Akron, USA; 2 Neurology, Northeast Ohio Medical University, Rootstown, USA; 3 Neurology, Corewell Health, Grand Rapids, USA; 4 Endovascular Neurology, Summa Health, Akron, USA

**Keywords:** intravenous thrombolytic therapy, mechanical thrombectomy in pediatrics, pediatric stroke, stroke, tenecteplase (tnk)

## Abstract

Although rare, stroke in children is a worrisome event that can have a profound impact on their development and future health outcomes as adults. While the use of the thrombolytics has become a mainstay of treatment for ischemic stroke in adults at comprehensive stroke centers, neither the efficacy nor the safety of thrombolytics for stroke in children has been established. This is, in part, due to the rarity of the pathology and difficulty in recruitment for clinical studies. Additionally, the more recently adopted thrombolytic, tenecteplase, has even fewer known cases of use in the treatment of ischemic stroke in children. We present a case of a 16-year-old male who presented with an ischemic stroke affecting the middle cerebral artery territory, which was promptly, safely, and successfully treated with tenecteplase.

## Introduction

Arterial ischemic stroke (AIS) in children is a serious cause of neurologic and intellectual disability. Current estimates of pediatric stroke incidence in the United States range from 1.3 to 5.4 per 100,000, with ischemic stroke making up ~60% of cases, and hemorrhagic stroke making up the remainder [1,2]. In comparison, adult stroke is much more common, with an incidence of 242 per 100,000 [3]. Stroke incidence in children is highest among neonates to four-year-olds and among 14- to 18-year-olds, with hemorrhagic strokes predominating the former group and ischemic strokes predominating the latter [2].

Unlike in adults, where the adoption of both thrombolytics and thrombectomy has transformed stroke care, the use of thrombolytics in children is less frequent, and the safety data on their use are scarce. Insufficient recruitment in randomized control trials of thrombolytics in children, such as in the Thrombolysis in Pediatric Stroke (TIPS) study, has led to this lack of safety data [4]. The most severe side effects of thrombolytic administration in order of severity are symptomatic intracranial hemorrhage (ICH), asymptomatic ICH, extracranial hemorrhage, and epistaxis. Notably, the retrospective study TIPSTER (TIPS Extended Results) conducted on children who had been treated with alteplase during the attempted TIPS study showed that no child experienced symptomatic ICH and only one experienced epistaxis following alteplase infusion [5]. Nonetheless, neither this nor other studies have had the statistical power to demonstrate complete safety in the use of thrombolytics in children for stroke.

Tenecteplase (TNK) achieves a prolonged half-life, increased clot-bound fibrin specificity, and increased resistance to plasminogen activator inhibitor-1 compared with alteplase [6,7]. Its administration as a single bolus, rather than a bolus-plus-infusion, simplifies its use and has been used for several decades as an FDA-approved thrombolytic in the treatment of acute myocardial infarction. It has been demonstrated in several randomized controlled trials, such as ATTEST, to be both noninferior to and as safe as alteplase in the treatment of ischemic stroke at a dose of 0.25 mg/kg up to 25 mg within 4.5 hours of symptom onset [8,9]. When combined with mechanical thrombectomy in eligible candidates, TNK is superior to alteplase for early reperfusion of occluded internal carotid, middle cerebral, or basilar arteries, as established by the EXTEND-IA trial [10].

In the case of the use of TNK in pediatric stroke, the paucity of safety and efficacy data has likely contributed somewhat to its low use. In a survey of pediatric neurologists worldwide, only 6% had cared for a child who received TNK, and only 21% had endorsed it being available on formulary at their pediatric hospital. However, 63% of respondents are willing to use TNK, with the most common reason for aversion to its use being the lack of efficacy data, lack of safety data, and unavailability [11]. Intending to bridge this gap in interest and usage, we present a case of a 16-year-old boy with an ischemic stroke who was successfully treated with TNK and demonstrated rapid improvement with no adverse effects.

## Case presentation

**Figure 1:**
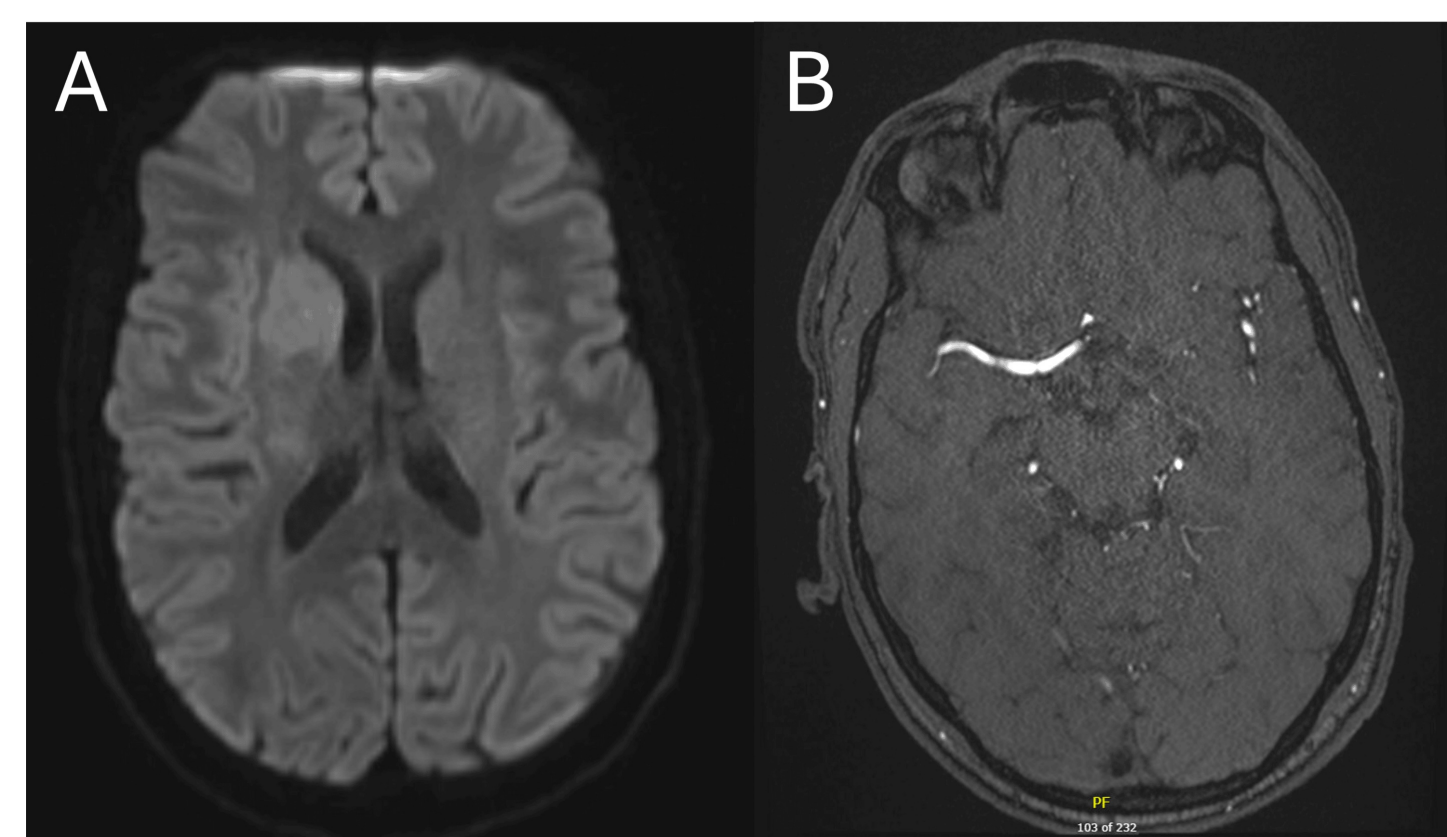
(A) Diffusion-weighted sequence from the initial MRI of the head at the level of the caudate. (B) Initial MRA at the level of the M2 segment of the MCA. (A) In the region of the right caudate head and anterior putamen, there is a diffusion restriction, which is a sign of an acute stroke when an MRI is performed. (B) The majority of the MCA is visualized on the right, with only one of the expected two M2 segments being clearly patent. The anterior M2 segment is occluded at the bifurcation of the M1 segment of the MCA into the M2 segments. MRA: Magnetic resonance angiography; MCA: middle cerebral artery.

At an emergency department of a pediatric academic center, a 16-year-old male presented 40 minutes after developing sudden-onset left hemiparesis and collapsing while shoveling snow. His past medical history included anxiety and attention deficit hyperactivity disorder (ADHD) for which he was taking citalopram, while his social history included extensive participation as a cross-country runner. He had no smoking or drug-use history, and his family history was noncontributory for coagulopathy, connective tissue disease, or early atherosclerosis. Within 30 minutes of arrival, an urgent MRI of the brain was ordered, and the imaging demonstrated a diffusion restriction in the right caudate head and anterior putamen (Figure 1A). A magnetic resonance angiography (MRA) of the head and neck was also performed, albeit delayed due to patient difficulty with remaining still and the need for sedation. It demonstrated a thrombosis of the right anterior temporal branch of the middle cerebral artery (MCA), corresponding to an M2 segment occlusion (Figure 1B). These findings prompted urgent transfer to an adult comprehensive stroke center and activation of a code stroke upon arrival.

**Figure 2:**
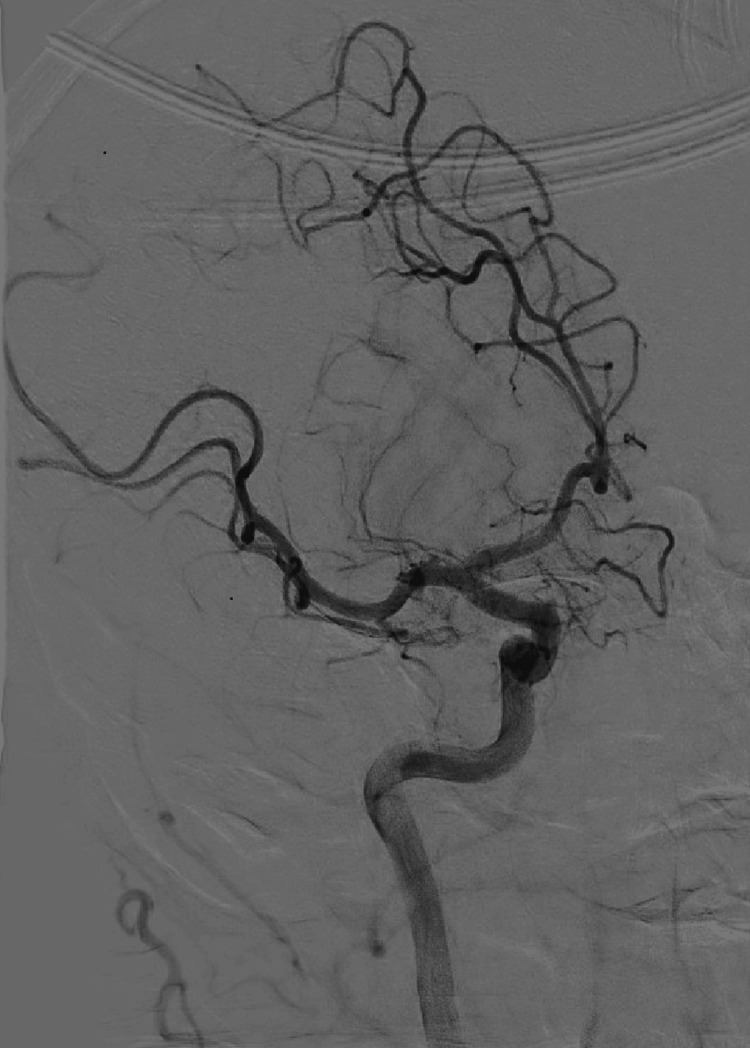
Cerebral angiogram of the internal carotid artery (ICA) and branches of the anterior cerebral artery (ACA) and middle cerebral artery (MCA). The right internal carotid artery is completely patent and gives rise to the completely patent ACA and MCA on the right.

Upon arrival at the adult center, the symptoms of left upper and lower hemiparesis were scored 8 on the National Institutes of Health Stroke Scale (NIHSS). CT of the head, CT angiography of the head and neck, and CT perfusion imaging were performed per protocol. CT was unremarkable for an acute bleed, while CT angiography redemonstrated the occlusion of the proximal M2 branch of the right MCA. CT perfusion showed a 40 mL mismatch (i.e., penumbra) with no ischemic component. Given that the last-known-well of the patient was about four hours from symptoms onset, hence within the 4.5-hour therapeutic window for TNK administration, and he had no contraindications to thrombolytics except for off-label use in the pediatric population, a thorough discussion of the risks, benefits, and alternatives of TNK was conducted with the patient’s parents, who agreed with its use. After TNK administration, the patient’s NIHSS score worsened to 11 with newly noted left-sided neglect, prompting transfer to the angiography suite for consideration of mechanical thrombectomy within 20 minutes. Cerebral angiography revealed reperfusion of the right MCA territory (Figure 2), leading to the premature cessation of the procedure with perioperative improvement of NIHSS score to 5.

**Figure 3:**
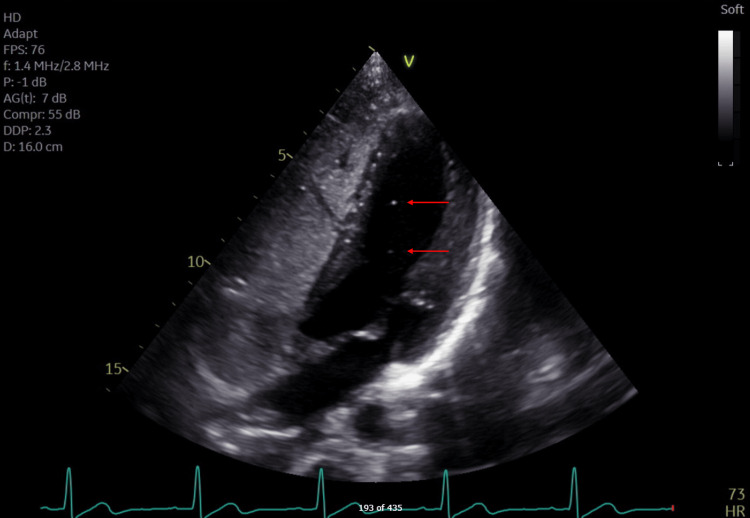
Transthoracic echocardiogram of the patient undergoing an agitated saline study. Complete opacification is noted in the right atrium and ventricle, signifying an adequate study, while two bubbles are noted with red arrows, signifying a Grade I positive agitated saline study.

He was subsequently transferred to the neurological intensive care unit and monitored closely. Twelve hours from his presentation, his symptoms had nearly resolved, with the only deficit being minor weakness of the left upper extremity and clumsiness in fine movements. An MRI was obtained after 24 hours to evaluate the extent of the stroke and showed no extension of the stroke territory. After being stable for over 48 hours, he was transferred to the general neurology ward and started on aspirin 325 mg for secondary stroke prophylaxis. Doppler ultrasonography of all four extremities was negative for any deep vein thromboses, while a transthoracic echocardiogram with an agitated saline study revealed a grade 1 patent foramen ovale (PFO) (Figure 3). He was discharged home in stable condition with plans for close follow-up and intensive physical rehabilitation. Physical rehabilitation occurred initially daily for one week, followed by two to three times weekly for two months, and included squats, sun salutations, and step-up exercises.

The PFO was percutaneously repaired two weeks following the hospitalization. Exploring the etiology of the stroke, a hematologic work-up included protein C and S activity, factor VIII activity, anti-phospholipid syndrome antibody testing, prothrombin mutation, B2 glycoprotein I antibody, C4 & C4 complement levels, and factor V Leiden. All these studies were negative, with the exception of an elevated factor VIII activity, which has limited clinical significance, given its nature as an acute-phase reactant. About six months following the acute stroke event, he has returned to cross-country running with minimal signs of the stroke having even occurred.

## Discussion

In this case, an acute AIS in a pediatric patient was successfully treated with TNK without an adverse outcome. This intervention was considered, given its known efficacy in adults and a close evaluation of the risks and benefits associated with treatment. In this instance, the adverse events that could have occurred include, most severely, a symptomatic intracranial hemorrhage, and less severely but still concerning, an asymptomatic intracranial hemorrhage, extracranial hemorrhage, including gastrointestinal hemorrhage, and other drug-associated reactions. As mentioned in the introduction, a detailed evaluation of the risks of these adverse events was attempted with the TIPS study, but was ultimately unsuccessful due to low recruitment [4].

Despite these risks, choosing supportive care over thrombolytic administration would likely have resulted in permanent cerebral ischemia and permanent disability of his left upper and lower extremities. The implications of this permanent disability would have drastically affected his ability to perform basic or instrumental activities of daily living (ADL), thereby diminishing his quality of life and leading to a lifelong need for costly ancillary care. Instead, following several months of physical rehabilitation, the patient has returned to normal functioning from a physical standpoint, even reinvolved himself in the physically intensive activity of cross-country running with minimal disability. Although it was not a direct consideration in our case, the ancillary costs associated with post-stroke care are substantial, and the reduction in these costs can be attributed to the level of disability upon discharge. Within the Kaiser Permanente Health system between 1996 and 2003, the post-admission chronic cost of pediatric stroke patients over five years with major neurologic deficits on discharge was $71,434, with a median of $25,551, while those with a near-normal neurologic examination on discharge had a mean cost of $52,604, with a median of $10,579 [12].

As had been established as non-inferior and safe by the ATTEST and EXTEND-IA trials, the dose of 0.25 mg/kg was selected for the treatment of this patient [9,10]. However, it is important to consider the difference in hemostatic capacity between them and adults. Children aged 11 to 16 years have been found to have reduced circulating procoagulants, such as factors II, V, VII, IX, X, XI, and XII, which were found to be reduced by 80% compared to adults. Similarly, there exist reductions in anticoagulants, including reduced amounts of antithrombin III to 60% of mean adult levels and protein C and S to 40% of mean adult levels [13]. Although the difference in procoagulant and anticoagulant levels is reduced, the asymmetry in their reduction could lead to a difference in how a pediatric patient responds to TNK, which makes applying adult dosages to pediatric patients difficult. Knowing these risks, the precautions taken to treat a pediatric stroke should at least mirror those of treatment in an adult, including the lowering of blood pressure to at least 185/110 prior to TNK administration to minimize hemorrhagic transformation risk, the monitoring of the patient in a critical care setting, the use of nursing staff trained in a standardized neurologic exam such as the NIHSS very frequently (i.e., q15 minutes for one hour, q30 minutes for two hours, and q1 hour thereafter), a low threshold for repeat imaging if a new neurologic deficit is detected, and ensuring the availability of cryoprecipitate or antifibrinolytics such as tranexamic acid in the event of an intracranial hemorrhage [14,15].

## Conclusions

Our case demonstrates a successful and favorable outcome following the use of TNK in a pediatric AIS and contributes to the limited literature regarding this topic. The patient’s rapid clinical improvement, as well as angiographic evidence of reperfusion, suggest that TNK may be a viable option in pediatric AIS in the absence of contraindications. This report adds to the emerging body of anecdotal evidence of successful administration of TNK for the treatment of AIS in children, but underlies the necessity for the establishment of registries or prospective studies that can closely evaluate the safety, efficacy, and long-term outcomes of TNK administration.
